# Supplementary material for: Can Citizen Science Contribute to Avian Influenza Surveillance?
Source: Pathogens. 2023 Sep 21;12(9):1183. doi: 10.3390/pathogens12091183 (PMC10535995; doi:10.3390/pathogens12091183)
Supplement: Supplementary file 1 [file pathogens-12-01183-s001.zip › pathogens-2513742-supplementary.pdf]

Supplementary material

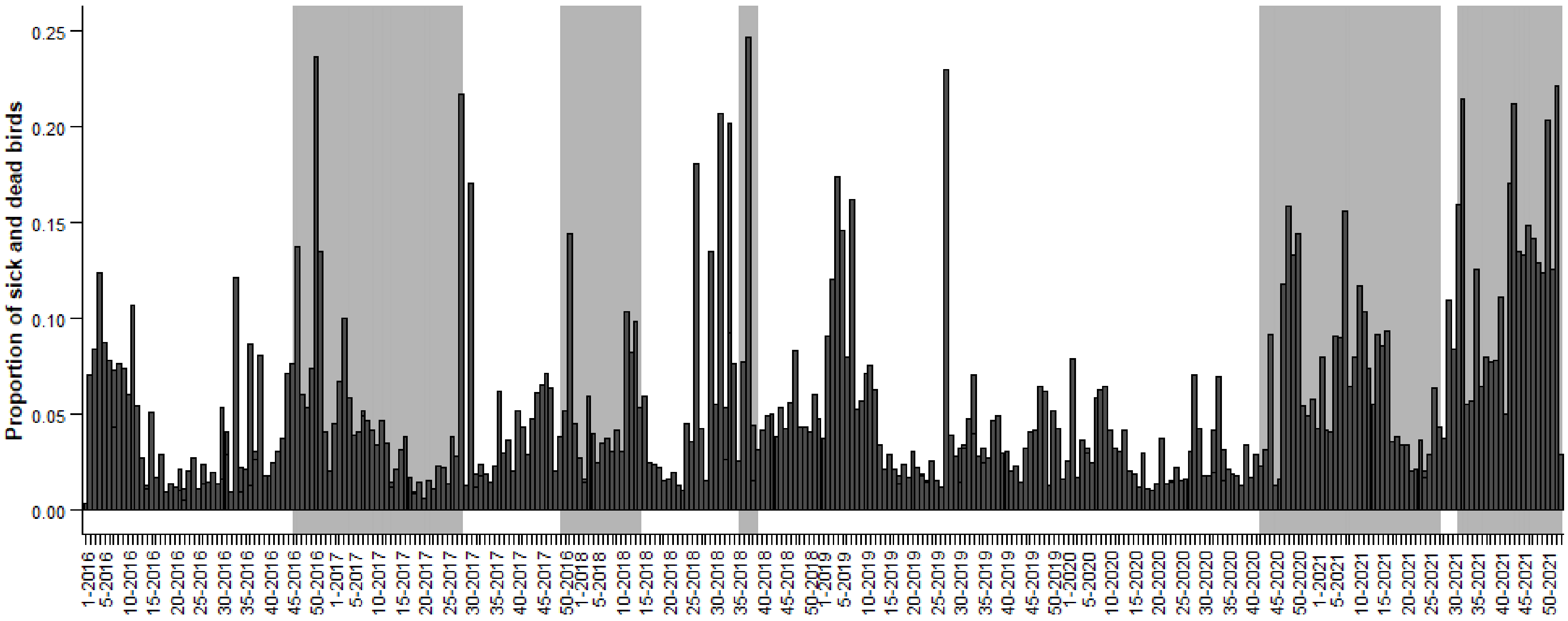

Figure S1. Temporal variation in the proportion of sick and dead birds by week (dark grey bars) and the periods of officially reported AIV outbreaks (light grey bars).

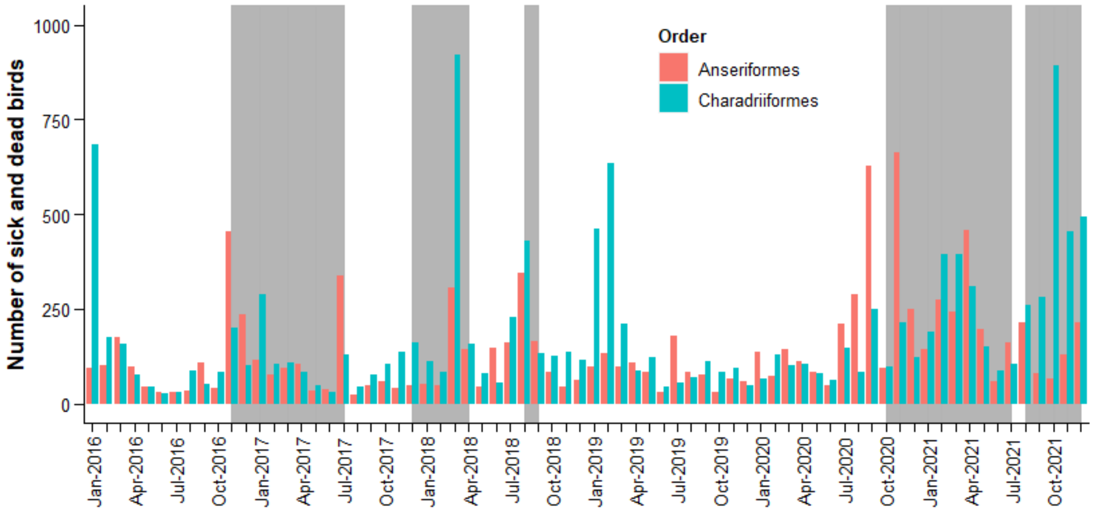

Figure S2. Temporal variation in the proportion of sick and dead birds of Anseriformes and Charadriiformes by month and the periods of officially reported AIV outbreaks (light grey bars).
